# Prophylactic Cancer Vaccines Engineered to Elicit Specific Adaptive Immune Response

**DOI:** 10.3389/fonc.2021.626463

**Published:** 2021-03-29

**Authors:** Davis W. Crews, Jenna A. Dombroski, Michael R. King

**Affiliations:** Department of Biomedical Engineering, Vanderbilt University, Nashville, TN, United States

**Keywords:** cancer, vaccine, adaptive immune response, prophylactic vaccine, biomaterials

## Abstract

Vaccines have been used to prevent and eradicate different diseases for over 200 years, and new vaccine technologies have the potential to prevent many common illnesses. Cancer, despite many advances in therapeutics, is still the second leading causes of death in the United States. Prophylactic, or preventative, cancer vaccines have the potential to reduce cancer prevalence by initiating a specific immune response that will target cancer before it can develop. Cancer vaccines can include many different components, such as peptides and carbohydrates, and be fabricated for delivery using a variety of means including through incorporation of stabilizing chemicals like polyethylene glycol (PEG) and pan-DR helper T-lymphocyte epitope (PADRE), fusion with antigen-presenting cells (APCs), microneedle patches, and liposomal encapsulation. There are currently five cancer vaccines used in the clinic, protecting against either human papillomavirus (HPV) or hepatitis B virus (HBV), and preventing several different types of cancer including cervical and oral cancer. Prophylactic cancer vaccines can promote three different types of adaptive responses: humoral (B cell, or antibody-mediated), cellular (T cell) or a combination of the two types. Each vaccine has its advantages and challenges at eliciting an adaptive immune response, but these prophylactic cancer vaccines in development have the potential to prevent or delay tumor development, and reduce the incidence of many common cancers.

## Introduction

Vaccines have improved the human condition since Edward Jenner developed the first vaccine to prevent smallpox over 200 years ago, paving the way for the prevention and even eradication of many common ailments (1). Cancer is the second leading cause of death in the United States, with 1.89 million people projected to be diagnosed with cancer in 2021 alone (2, 3). While there are many successful therapeutics for cancer treatment, advances in prophylactic vaccination against cancer have been limited.

Preventative, or prophylactic, cancer vaccines have the potential to reduce cancer prevalence and improve prognosis by inducing an immune response to prevent the development of specific cancers. Currently, five vaccines are used in clinical practice and approved by the FDA. These vaccines protect against two cancer-promoting viral infections, hepatitis B (HBV) and human papillomavirus (HPV) (4). HPV is a sexually transmitted infection, with several of its forms associated with different types of cancers, the most common being cervical and oral cancer. Individuals vaccinated with the HPV vaccine are protected from cancer by preventing HPV development; since HPV can promote the onset of cervical cancer, HPV prevention is expected to lead to its decline (4). In Scotland, women vaccinated for HPV showed an 89% reduction in cervical intraepithelial neoplasia (CIN) grade 3 or worse when compared to non-vaccinated women. Similar reductions were shown in CIN grade 1 and grade 2 (5). Furthermore, vaccination against HBV, a risk factor for hepatocellular cancer, of infants in Taiwan has shown reduced cancer prevalence. Rates of hepatocellular cancer in vaccinated Taiwanese children age 6-14 years fell approximately 70% (4).

**Figure 1:**
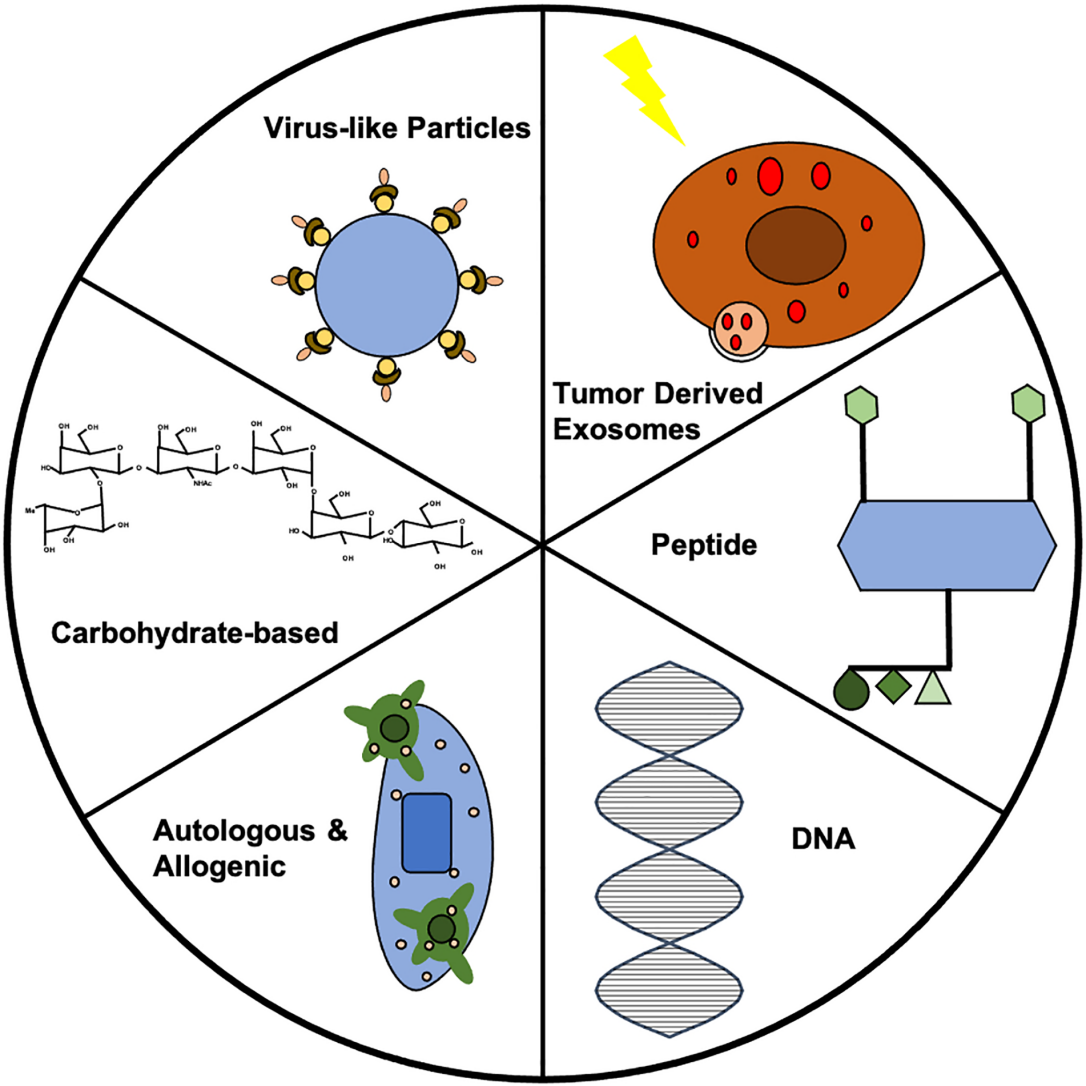
Summary of vaccine strategies. Each vaccine strategy has potential for use in prophylactic cancer vaccines. Further investigation into each strategy could lead to clinically-relevant prophylactic cancer vaccines. In this figure, the VLP represents the HER2-VLP, which has elevated levels of anti-HER2 antibody to protect against breast cancer. The carbohydrate displayed is the chemical structure of Globo H, which has often been used in therapeutic vaccines, but shows potential for prophylactic vaccine development. The allogenic vaccine displays dendritic cells (DCs) recognizing the tumor antigen, which can allow for immune cell activation. The double helix of DNA is the building block for all DNA vaccines. The peptide vaccine shows four epitopes engineered for display, which caused upregulation of CD4+ and CD8+ T cells in addition to increases in IgG antibodies in vaccinated mice. The exosome shows TEX synthesis *via* radiotherapy that prevented breast cancer *via* CD8+.

While preventative vaccines are commonly implemented, preventative vaccines designed to protect against cancer are a relatively new development. The goal of preventative cancer vaccines is not to treat, but to prevent the development of a tumor. Cancer vaccines are often defined as therapeutic vaccines, which are different from prophylactic vaccines in that they elicit an immune response to an existing tumor and to residual cancer cells following other treatments (6). Therapeutic vaccines against cancer elicit immune responses following the onset of disease. For example, proposed therapeutic vaccines against breast cancer can target human epidermal growth factor receptor 2 (HER2), utilizing T cells to elicit a targeted immune response (7). While strides are being made in therapeutic vaccines for cancer, many different strategies have been proposed for the development of prophylactic cancer vaccines (**Figure 1**).

Preventative vaccines offer many advantages over therapeutic treatments in terms of health and cost benefits. Preventative treatments reduce morbidity and mortality, and current vaccines from childhood vaccines like Tdap (Tetanus, diphtheria, pertussis) to influenza vaccines have led to economic benefits in low- and middle-income countries (8). Successfully engineered cancer vaccines could decrease health care costs associated with cancer. It has been estimated that the total cost of cancer in the United States would increase 34% in just fifteen years, from $183 billion in 2015 to $246 billion in 2030 (9). Thus, decreasing the cost of cancer treatments through preventative vaccines could result in dramatic decreases in healthcare costs associated with cancer.

Vaccines have demonstrated the ability to successfully eradicate previously common diseases, controlling the spread of 12 diseases, such as smallpox and yellow fever (10). Disease eradication is a common and efficient way to improve public health (10). The successful development of preventative cancer vaccines could decrease the prevalence of cancer, reducing cancer-related deaths. HPV vaccines have already reduced the prevalence of cervical cancer. In Scotland, an 89% reduction in grade 3 or worse cervical intraepithelial neoplasia (CIN) was seen for women vaccinated at 12-13 years old (5). Cervical cancer prevention with HPV vaccines provide promise that vaccines can be developed for other cancers to achieve similar results. Several possible strategies for cancer prevention will be discussed in this review, with each presenting distinct advantages and challenges (**Table 1**). Age-related immune decline is seen across all vaccine engineering strategies as a major challenge.

**Table 1:** Advantages and disadvantages of different prophylactic cancer vaccine strategies currently being investigated.

Vaccine Strategy	Advantages	Disadvantages
Virus-like Particles	Overcome B cell tolerance (11) Humoral and cellular responses (12)	Must be highly stable for proper downstream applications (13)
Carbohydrate-based	Ease of synthesis (14) target unique glycans (15) Humoral and cellular responses (16)	Poor immunogenicity (17)
Peptide	High stability against degradation *in vivo* (18) Ease of synthesis (19) Humoral and cellular responses (20, 21)	Inefficient immune response (22)
Lipid Nanoparticle	Overcome genetic material degradation (23) Easily synthesized (24)	Difficult to evaluate and predict *in vivo* effectiveness to identify proper dosage and side effects (24)
DNA	Stable at ambient temperatures (25) Ease of preparation (25) Humoral and cellular responses (26)	Inadequate immunogenicity (27)
Tumor-Derived Exosomes	Play natural role in tumor progression (28)	Primarily cellular response (29)
mRNA	Low manufacturing cost (30) Potential high potency Possible non-invasive administration (31)	*In vivo* stability (30) Primarily cellular response (31)
Autologous Tumor Cell	Personalized formulations (32) Humoral and cellular responses (33)	Requires patient tumor cells (32) Mainly therapeutic currently (34)
Allogenic Tumor Cell	Clinical trials for therapeutic version (35) Humoral and cellular responses (36, 37)	Limited current effectiveness (38) Mainly therapeutic currently (38)

### Role of the Immune System in Cancer

A unique aspect of cancer is its ability to survive in the presence of an immune system, making immunotherapy a challenging yet promising therapeutic for cancer. This property stems from two essential hallmarks of cancer: tumor-promoting inflammation and avoiding immune destruction (39). Tumors utilize the immune system by generating an inflammatory response conducive to tumor growth (39). The tumor microenvironment (TME) consists of neoplastic tissue, which is highly disorganized and grows uncontrollably (40). Neoplastic progression is supported by inflammation of cytokines like interleukin-1 (IL-1) and tumor necrosis factor (TNF) (41, 42). Necrotic cells within the TME stimulate proliferation of neighboring cells through the release of IL-1α, and angiogenesis is driven by IL-1β (43, 44). IL-18 induces vascular cell adhesion expression, supporting invasion and metastasis (45). TNF-α promotes tumor development by regulating factors such as cytokines, chemokines, adhesion molecules, and matrix metalloproteinases (MMPs) (46).

Tumors evade immune detection, and therefore destruction, through a variety of means including regulatory cells, down-modulating antigen presentation, tolerance, and immune suppression (39, 47). Not only does the hypoxic tumor promote regulatory T cell (Treg) homing to the TME, but tumor-derived CD4+CD25+FoxP3+ Tregs have been found to be more suppressive of cytotoxic lymphocytes (CTLs) than normal Tregs (48–50). Tumors are able to evade CTL recognition by down-modulating essential components of antigen processing and presentation such as the MHC I pathway (47). Tolerance is induced by tumor cells, since they do not express co-stimulatory molecules that are needed to activate T cells or antigen-presenting cells (APCs) (51). Furthermore, CTLA-4 and PD-1 are upregulated on cancer cells, inhibiting a T cell response (52). The combination of these various traits of cancer contribute to the difficulty of the immune system to independently stop tumor development, making a prophylactic vaccine a useful approach for cancer prevention.

### Cancer Vaccines and the Immune System

The goal of a prophylactic cancer vaccine is to elicit an adaptive primary immune response, to allow for a rapid and strong secondary response if carcinogenesis occurs (53, 54). The mechanism behind these preventative vaccines can be viewed as specific immunity to modified self-antigens, therefore producing an immune response to cells that have undergone malignant transformation (55). Cancer vaccines can be developed to recognize and prevent cancer-promoting viruses or neoantigens, which are peptides found on tumor cells that are associated with spontaneous cancers (56).

**Figure 2:**
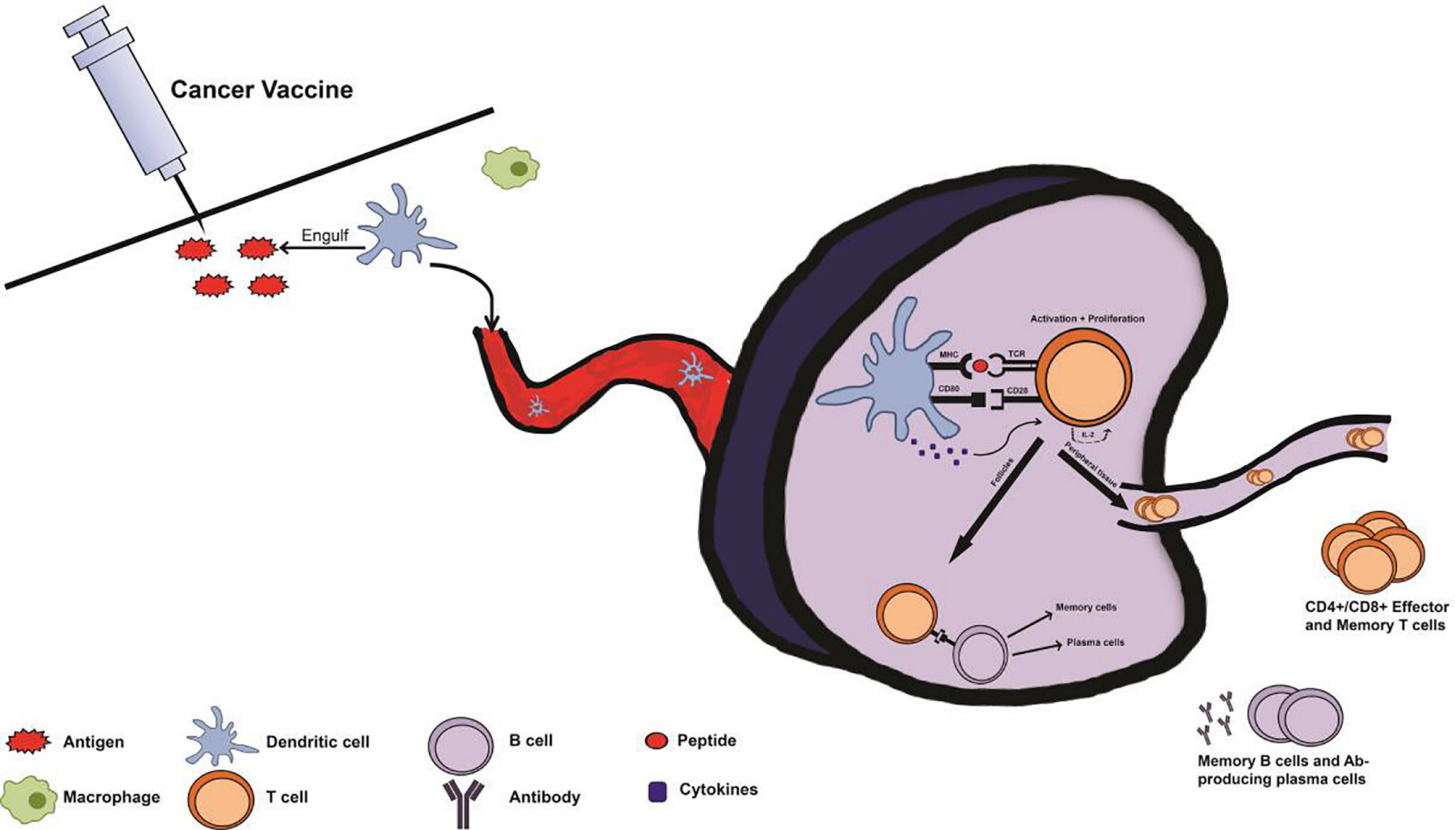
Immune system response with prophylactic cancer vaccine administration. Following administration of a cancer vaccine, antigen-presenting cells (APCs) from the innate immune system such as macrophages and dendritic cells (DCs) recognize the injected antigen as foreign *via* pattern recognition receptors (PRRs), and uptake the antigen. Subsequently, the APCs transport the antigen, migrating to a lymph node and processing and presenting the antigen *via* major histocompatibility complex (MHC) pathway. Once in the lymph node, an immune synapse will form as the APC presents the antigen to an immature T cell at the T cell receptor (TCR). T cells will be activated by this interaction, with the aid of cytokines and co-stimulatory signals from the APC. Upon activation, T cells proliferate *via* IL-2 production and differentiate into effector T cells depending on cytokines and MHC type from the APC. These T cells can then contribute to the activation of B cells or travel to distant sites as effector or memory T cells. This primary response following vaccination produces memory cells so that secondary exposure to cancer-associated antigens results in a rapid and robust secondary immune response.

Microbes and other foreign bodies included in a vaccine alert the host immune system *via* presentation of Damage-Associated Molecular Patterns (DAMPs), which cause innate immune cells such as APCs to produce cytokines necessary for activating T cells (**Figure 2**). This activation can result in the production of effector or memory T cells, or facilitate the activation of B cells, ultimately leading to lasting immunity (53). By producing this adaptive response, a vaccine develops memory for protection from an antigen (54). Adaptive immune responses can consist of T cell-mediated cellular responses, B cell-mediated humoral responses, or combinations of the two (57). By activating T and B cells, a vaccine is able to produce memory T and B cells, which are essential for stopping another attack or antigen exposure (57). These memory cells proliferate, causing a stronger, faster response upon a second exposure (54).

While there are many preventative cancer vaccines being developed in the research setting, there are only five clinically-relevant cancer vaccines, three for HBV and two for HPV (4). These vaccines are successful because they avoid major issues in the development of a therapeutic vaccine: an immunosuppressive tumor microenvironment, low immunogenicity of the antigen, and a disease with high incidence (58). Successful prophylactic cancer vaccines take advantage of the immune system to provide lasting benefits of cancer prevention. Cancer vaccines can be used to prevent the formation of virally onset cancers or spontaneous cancers by initiating immune responses against a virus or neoantigen target.

### Safety Concerns and Challenges of Prophylactic Cancer Vaccines

It is essential that preventative vaccines, given to healthy patients, do not cause any adverse side effects such as an autoimmune response (59). Therefore, high risk individuals—those with increased risk of a specific cancer—are often the best candidates for such vaccines (59). Patients with syndromes such as hereditary non-polyposis colorectal cancer (HNPCC) have a genetic predisposition for specific cancers, motivating the development of a viable preventive measure (60). Since many of the prophylactic vaccines developed involve unnecessary exposure to cancer antigens, these vaccines must be engineered to ensure antigens do not increase cancer risk. This could also pose a problem for public acceptance and successful implementation of prophylactic cancer vaccines into the clinic. Other safety concerns include off-target effects and toxicity related to any possible vaccine materials (61). Successful engineering of prophylactic vaccines must consider the issue of safety earlier in development than therapeutic vaccines, as preventative vaccines are intended for healthy individuals.

There are several challenges that prophylactic vaccines must overcome that are described in this review. These obstacles include poor immunogenicity of common vaccine formulations, and poor stability *in vivo*. Furthermore, prophylactic vaccine trials may need to be proceeded by therapeutic vaccine development, as vaccine dosages for healthy clinical trial participants must be low. Immune system decline in elderly patients is another challenge faced by prophylactic cancer vaccines, as adaptive immunity is paramount to vaccine success. With 70% of cancer-related deaths occurring in patients 65 and older, this poses a significant problem that must be addressed (62).

## Humoral Cancer Vaccines

Humoral, or antibody-mediated, vaccines invoke B cell responses to prevent disease and have the ability to last for decades, a goal of preventative cancer vaccines. For instance, smallpox vaccines can cause the maintenance of vaccinia-specific IgG+ memory B cells for more than 50 years (63). Another benefit of humoral vaccines is the possibility for secondary tumor antigen targeting. One phenomenon, known as epitope or antigen spreading, is an important concept in vaccine development, where an immune response develops for epitopes that are different than the disease-causing epitope, allowing for more complete and robust protection from disease (64, 65). Studies have shown epitope spread can increase the effectiveness of previously developed therapeutic cancer vaccines (66). For example, the FDA approved therapeutic autologous immunotherapy vaccine for metastatic castration-resistant prostate cancer, Sipuleucel-T, facilitates T cell priming and also results in elevated levels of antigen spread. This results in higher levels of IgG against secondary tumor antigen, increasing overall survival (64). Epitope spread has also been associated with tumor regression (67). In one study, highly specific intramolecular epitope spreading was partly responsible for preventative effects of a vaccine against KRAS-induced lung cancer (68).

Humoral vaccines offer other advantages in the form of neutralization and antibody-dependent cellular cytotoxicity (ADCC). These mechanisms protect cells from viral infection instead of controlling previously infected cells. Neutralizing antibodies function by binding to the virus, alerting the immune system to the presence of a foreign body and preventing the virus from infecting a cell (69). Antibody neutralization of the virus HPV can prevent infection by multiple mechanisms, such as prevention of cell surface binding and disruption of virus internalization (70). Humoral protection *via* neutralization of oncoviruses is an effective strategy to prevent some cancers, such as cervical cancer. ADCC, which utilizes innate immune cells to provide antitumor activity by linking antibodies to target cells, is also a vital part of the humoral response. Natural killer cells play a major role in ADCC, as they are responsible for provoking the immune response and direct cytotoxicity of cells infected by a virus and tumor cells (71). One study found that the success of a preventative human immunodeficiency virus (HIV) vaccine could be partially attributed to an ADCC response (72). Several cancer vaccines, including a MUC1-based cancer vaccine, have successfully elicited an ADCC response (73).

Verifying successful humoral response requires accurate quantification of antibodies and protein expression in patient plasma and tissue samples. Research has shown that many patients have a natural immunity to mesothelin, a glycoprotein expressed in several common cancers. ELISA analysis of IgG antibodies in patient serum and immunohistochemistry analysis of mesothelin protein expression of tumor specimens can be used to evaluate the effectiveness of vaccines and find potential antigen targets (74). A significant disadvantage of humoral vaccines stems from elderly patients having particularly weak humoral immune responses. Aging is associated with decreased B cell levels, which are essential for humoral vaccine success. *In vitro* and *in vivo* studies on tetanus toxoid showed decreases in IgG secretion in elderly patients, with younger patients not only having more B cells, but more potent B cells (75). Following immunization, younger patients also displayed elevated IgG levels for up to a year, while elderly patients returned to baseline levels after only 6 months (75). Since the age distribution of cancer patients skews toward the elderly, this is particularly concerning for the development of humoral cancer vaccines. Thus, when engineering cancer vaccines, especially for cancers originating from the lung, prostate and colon, which are common in elderly patients, it is vital to consider decreased humoral response (76).

### Virus-Like Particles

Virus-like particles (VLPs) are protein structures with multiple subunits (13). Several VLPs have been engineered to prevent cancer by eliciting a humoral immune response, often through increasing IgG levels. In the past, VLPs have been used to treat many different viruses, as VLPs closely resemble the structure of the virus it is being used to prevent but lack virus-specific genetic material (13, 77). Specifically, commercially available vaccines against HPV are VLP-based, including Cervarix and Gardasil, in addition to the HBV vaccine (78). However, stability remains a large concern for VLP vaccine development, as VLP vaccine success depends on downstream effects leading to a need for high stability (13). VLPs have been investigated to treat viruses. Future VLP vaccines must consider the issue of safety earlier in development than therapeutic vaccines, as preventative vaccines are intended for healthy individuals.

A VLP vaccine has been engineered to treat and prevent human epidermal growth factor receptor-2 (HER2)-positive breast cancers (11). To synthesize this VLP, S2 insect cells were transfected to express SpyCatcher-HER2 fusion antigen and incubated with Spytagged VLPs for a stable antigen coating. Transgenic mice, which can spontaneously develop HER2-positive mammary carcinoma, were vaccinated with this HER2-VLP. Vaccinated mice showed no tumor growth until one year of age, while untreated mice developed tumors after only two months. Elevated levels of anti-HER2 antibody were found in vaccinated mice for at least 24 weeks (11). HER2-VLP induced a stronger antibody response and provided better protection against tumor onset than a previously studied DNA vaccine, which had been more effective than passive administration of trastuzumab in HER2 transgenic mice. Furthermore, the anti-HER2 antibodies induced by the vaccine showed comparable affinity to that of monoclonal antibody (mAb) trastuzumab, a HER2-targeting antibody, and the vaccine showed decreased tumor onset when compared to mAb trastuzumab passive administration. The HER2-VLP vaccine inhibited not only tumor onset, but tumor growth, suggesting both preventative and therapeutic effects achieved by the vaccine (11).

Importantly, the HER2-VLP vaccine overcame B cell tolerance, a phenomenon which occurs when B cells die to prevent autoreactive antibody synthesis and is a frequent obstacle for humoral vaccine development (79). VLP vaccines can overcome this issue since they exhibit multivalent display of self-antigen (11). One study demonstrated that multivalent VLP induced higher IgG titers and overcame the effects of anergy (80). This outcome is likely due to VLP multivalency increasing the ability to create stable signaling domains, causing an increase in B cell activation (80). Using anti-HER2 antibodies from the mice, IgG antibodies elicited strong binding to HER2-positive human tumor cell lines, but no binding was detected on HER2-negative cell lines.

### Carbohydrate-Based Vaccines

Using carbohydrate structures to induce an immune response is a promising direction in the field of vaccines (15). Cell-surface glycans are targeted by carbohydrate-based vaccines (15). While most carbohydrate-based vaccines are currently limited to therapeutics for infectious diseases, applications for preventative cancer vaccines have been proposed and studied (15). Specifically, the hexasaccharide Globo H (GH) has been proposed to both treat and prevent cancer (81). Globo H is a carbohydrate located on the outer membrane of epithelial cells and is often overexpressed in a variety of tumor specimens, including breast, ovarian, and lung cancer (82). Huang et al. proposed synthesizing a GH and linking it to a carrier protein as a therapeutic treatment for SSEA4-expressing breast cancers (81). Although this is strictly therapeutic, mice treated with this vaccine elicited IgG antibodies against the SSEA4 ganglioside, which can be overexpressed in breast cancer (81).

GH can be synthesized *via* glycal chemistry, one-pot synthesis or enzymatic synthesis. Among these methods, enzymatic synthesis is the cheapest and easiest, and requires enzymes overexpressed in *Escherichia coli* (14). Using sugar nucleotide regeneration and glycosyltransferases, GH can be synthesized in just three steps (14). The GH vaccine engineered by Danishefsky et al. not only induces anti-GH antibodies, but also anti-SSEA3 and anti-SSEA4 antibodies. These three glycoproteins are overexpressed on over 16 cancer types (83). Additionally, a glycolipid adjuvant was designed, which targeted CD1d receptors found on dendritic and B cells to cause a shift to IgG production. This process induces a switch from IgM, which is usually the sole response induced by carbohydrate-based vaccines (83). While this vaccine functions currently as a therapeutic, Danishefsky et al. indicate the possibility of using this vaccine in a preventative manner. The proposed design lays a framework for successful engineering of future carbohydrate-based vaccines. Unique glycan markers associated with cancer can be identified for use as a target, and then a carbohydrate compound can be designed using chemical and immunological processes to effectively leverage the target for cancer prevention (83).

A common problem with carbohydrate vaccines is poor immunogenicity of tumor-associated carbohydrate antigens (TACAs). Sialyl-TN (STn) is an oncofetal antigen found in specific cancers and has been used as an adjuvant to boost the immunogenicity of TACAs (17). One study couples three fluoro-substituted STn analogues to the metalloprotein keyhole limpet hemocyanin (KLH). Fluorine-modified STn compounds can be used to increase immunogenicity and thereby increase the strength of the immune response (84). Previously, it has been shown that 4-KLH, a fluorine-modified STn antigen, results in increased IgG levels when compared to anti-modified-STn (85). Both therapeutic and preventative effects were observed *in vivo*. 4-KLH-vaccinated mice inoculated with colorectal cancer showed increased anti-STn antibodies when compared to a 1-KLH vaccine. 4-KLH also showed some preventative effects when compared to the control (84). This result could provide the framework for STn-KLH vaccines as a means to prevent cancer formation when used with the proposed fluorine modification strategy.

### Lipid Nanoparticle Vaccines

Lipid nanoparticle (LNP) vaccines have the potential to effectively deliver genetic information for cancer prevention. Delivery of mRNA and DNA to the body has potential to prevent cancer, but degradation is often a problem for delivery of naked genetic material (23). Use of LNPs can help overcome these problems for preventative cancer vaccines. LNPs are easily synthesized and can protect mRNA or DNA from degradation (24). However, there are challenges associated with LNP vaccine development. Assays to effectively predict *in vivo* responses do not currently exist, as current assays only measure second-order effects. LNPs may complete their goal successfully, but if certain pathways are not activated, these effects will be undetectable to current assays. This presents challenges when evaluating different formulations, dosages, and side effects (24).

## Cellular Cancer Vaccines

Cellular vaccines induce CD8+ and CD4+ T cell activity (57). For many successful vaccines, memory T cell induction is vital to eliciting a sufficient immune response to stop disease formation. This response requires large-scale changes in both the properties and number of T cells (86). The idea for cellular vaccines against cancer originated from successful T cell-mediated vaccines for viral infections. T cell-mediated vaccines can have both preventative and therapeutic benefits. For example, a vaccine engineered to prevent HPV and cervical cancer development induces CD8+ T cells, which provides lasting protection against HPV and associated diseases (87). While engineering new vaccines, it is important to consider that a sufficient dose is required to induce a T cell response strong enough to prevent disease, so a high dosage must not be toxic. Another concern with engineering cellular vaccines is overexertion of T cells (88). An overexerted immune system can cause T cell exhaustion, and ultimately, dysfunction. T cell exhaustion is the result of sustained expression of inhibitory receptors, low effector function, and an altered transcriptional state. It leads to decreased immune control of tumors and infections, and poor memory formation (88). Exhaustion can occur during chronic infection and cancer, making it a significant problem that must be addressed when engineering cellular vaccines against cancer (88). Another concern with cellular vaccines, as with humoral vaccines, is age-associated decline. T cell-mediated immunity declines with age due to alterations in the thymus, signal transduction and HLA Class II expression on monocytes (89–91). Aging is also associated with decreased T cell reactivity to foreign antigens (89). Despite these concerns, the successful engineering of preventative cellular vaccines against oncogenic viral infections, which cause 15% of cancers worldwide, offers promise for similar solutions to cancer prevention (92).

### Peptide Vaccines

Peptide vaccines use engineered short peptide fragments to induce a specific immune response (22, 93). Longer amino acid chains may also be used, but shorter chains are most common (93). Peptide vaccines can be engineered to have stability against degradation *in vivo* and are cost effective and easy to synthesize (18, 19). Nevertheless, they suffer from poor immunogenicity (22). A peptide vaccine designed to prevent breast cancer was formulated with stabilizing chemical pan DR epitope (PADRE), a carrier epitope used to engineer synthetic and recombinant vaccines (94). A nanoliposomal vaccine was designed using DOPE-containing liposomes and engineered with three different peptides (AE36, E75, and E75-AE36) used in combination with PADRE. Vaccinated mice showed higher CD4+ and CD8+ T cell induction when compared to mice treated with liposomal short peptides without PADRE and mice treated with non-liposomal peptides. Furthermore, increased IFN-γ levels were observed, which promotes adaptive immune mechanisms (95, 96). IFN-γ also plays a role in promoting tumor surveillance, although the exact mechanism is unknown. Previous studies have hypothesized that IFN-γ may even be the basis for immune surveillance. Nevertheless, it is clear that IFN-γ plays a role in directing tumor surveillance to chemically-induced tumors, as well as tumors caused by genetic defects (96).

Transmembrane protein GP2 has also been explored for use in peptide vaccines (97). GP2 vaccines have been explored as viable means to prevent breast cancer reoccurrence for HER2/*neu*+ patients. A polymorphism leading to a mutant GP2 protein called 2VGP2 was found at codon 655 of the HER2/neu protein and has been identified as a common mutation associated with higher risk of breast cancer (98). Autologous DCs from blood samples from HLA-A2 breast cancer patients were pulsed with synthesized GP2 and used to stimulate T cells *in vitro*. Cytotoxicity experiments showed killing of breast and ovarian cancer cells *via* GP2-stimulated CD8+ T cells. These experiments confirm GP2 immunogenicity and show its potential as a peptide vaccine against HER2/*neu*+ breast cancer (99).

A KRAS-targeting peptide vaccine has been engineered to prevent lung cancer. KRAS is considered a proto-oncogene, with mutant KRAS a common driver of cancer (100). A KRAS peptide vaccine was developed with four peptides corresponding to different regions of the protein, and CpG, R848, and anti-CD40 were used as adjuvants. This vaccine increased IFN-γ and granzyme B levels in CD8+ T cells, and when combined with avasimibe, resulted in infiltration of CD8+ T cells in tumors and prevented KRAS-driven lung tumorigenesis. Thus, this vaccine may be a starting point to develop vaccines to prevent premalignant lung legions with mutant KRAS from progressing to malignant lesions (101).

### DNA Vaccines

DNA vaccines are appealing due to the ability to mimic natural infections, ease of production, and stability at ambient temperatures (25). Several DNA vaccines have been developed to prevent prostate cancer but have had mixed results in clinical trials, DNA vaccines often failing due to inadequate immunogenicity (27, 102). A DNA vaccine was proposed to prevent castration resistant prostate cancer (CRPC), using RALA/pPSCA nanoparticles (NP) incorporated into a dissolvable microneedle (MN) patch. RALA codes for Ras-related protein Ral-A, a protein implicated in several cancers, and pPSCA is a plasmid encoding prostate stem cell antigen. RALA/pPSCA-loaded MNs caused endogenous production of prostate stem cell antigen, and induced a response against TRAMP-C1 tumors *ex vivo* and anti-tumor immunity *in vivo*. In prophylactic experiments, unvaccinated mice developed palpable tumors within seven days of tumor implantation, whereas vaccinated mice showed delayed tumor growth. On average, tumor development took 16.2 days for RALA/pPSCA-loaded MNs-treated mice, with one mouse remaining tumor free through the duration of the experiment. This study shows that the use of microneedles to administer a DNA vaccine could be a promising strategy to prevent cancer formation (26).

### Tumor-Derived Exosomes

Exosomes are microvesicles released by cells in physiological and pathological settings; exosomes are enclosed by a lipid bilayer with proteins from the origin cell. These cargo exosomes can assist in tumor progression and cancer metastasis by delivering parental cell proteins and nucleic acids to target cells (28). The proteins or nucleic acids in cargo exosomes that contain antigens associated with the parental cancer cells could, therefore, become targets for prophylactic vaccination. Tumor growth is promoted by tumor-derived exosomes (TEX), exosomes released from tumor cells. They signal to both cancerous and normal cells throughout the body and play a role in cancer progression (103). A vaccine against breast cancer has been engineered using TEX. TSA, a BALB/c mouse-derived mammary carcinoma, was exposed to Sham radiotherapy (RT) to develop TEX (104, 105). A vaccine of TEX from untreated cells (UT-TEX) was also used. The RT-TEX vaccine induced a tumor-specific CD8+ response, with 2 of 6 mice vaccinated showing no tumor growth and 4 showing reduced tumor growth compared to UT-TEX-vaccinated mice. RT-TEX-vaccinated mice had a higher number of CD8+ T cells in the tumor, many of which were specific to an immunodominant antigen in the tumor. This supports the idea that TEX produced *via* irradiated cancer cells is a viable strategy for cancer prevention (29).

### mRNA Vaccines

mRNA vaccines have the advantage of low-cost manufacturing and potential for high potency. However, stability *in vivo* is a large concern for successful engineering of mRNA vaccines against cancer (30). Even so, mRNA-based vaccines independent of VLP carriers are in development. Previous methods have injected DCs transfected with mRNA with promising results, but this method is costly (106). Another study recommended nasal administration of an mRNA vaccine for the prevention of cancer. Nasal administration is promising due to its non-invasive format and high patient compliance. Tests were performed for nasal administration of naked and nanoparticle encapsulated mRNA for tumor prevention using a mouse model. The mRNA was encoding for a tumor antigen. While the naked mRNA administration did not prevent tumor growth, nasal administration of mRNA encapsulated in nanoparticles was effective for tumor prevention. Therapeutic effects were also observed in additional experiments. Splenocytes recovered from the mice revealed anti-cancer CD8+ T cells in mice treated with the encapsulated mRNA vaccine. As one of the few mRNA vaccine studies available for cancer vaccination, this study shows possible effectiveness of mRNA vaccines for cancer prevention in addition to showing possible effectiveness of nasal administration of prophylactic and therapeutic cancer vaccines (31).

## Combined Cellular and Humoral Cancer Vaccines

As discussed above, there are advantages to both humoral and cellular vaccines. However, many vaccines induce both a humoral and cellular immune response. Vaccines can cause a biased immune response toward one type of adaptive immunity while inducing both T and B cell immunity (36). The benefit of a combined humoral and cellular response can be seen in many non-cancer vaccines. For instance, for influenza prevention, the trivalent live attenuated influenza vaccine (LAIV) induces both B cell and T cell responses. Conversely, the trivalent inactivated influenza vaccine (TIV), which only invokes a T cell response, has been found to be immunologically inferior (20).

### Combined Peptide Vaccines

While many peptide vaccines induce only a cellular immune response, others can induce both humoral and cellular responses. A mimotope peptide-based vaccine was developed using BAT monoclonal antibodies, which have immune modulatory and anti-tumor effects (20). Mimotopes are peptides that can bind to an antibody directed against a certain antigen (21). For this vaccine, BAT-binding peptides A and B were used as mimotopes. Vaccinated mice displayed increased IgG antibody production, which competed with BAT binding on Daudi cells, a human B lymphoblast. The IgG antibodies caused similar immune stimulation to BAT, indicating a humoral component to the vaccine. The observed cellular response included increased cytolytic activity, and the vaccine prevented tumor growth *in vivo* in a mouse model (20).

A self-adjuvanting multivalent glycolipopeptide (GLP) has also been developed as a vaccine (21). The GLP vaccines display four components on a molecular delivery system: TACA B cell epitope, CD4+ Th peptide epitope, CD8+ CTL peptide epitope, and immunoadjuvant palmitic acid. This GLP vaccine was administered in combination with PADRE and regioselectively addressable functionalized template molecules (RAFT). *In vivo*, vaccinated mice did not develop tumors over a 90-day period, while unvaccinated mice developed tumors around 35 days after tumor inoculation. Serum collected from BALB/c mice showed IgG antibodies specific to breast cancer, and upregulation of CD4+ and CD8+ T cells, indicating both humoral and cellular responses (21).

Another combined peptide vaccine has undergone clinical trials for the prevention of colorectal cancer. A MUC1-poly-ICLC vaccine has been tested for patients with advanced adenomatous polyps, which are a precursor to colorectal cancer. MUC1 is a glycoprotein and tumor-associated antigen (TAA) for colorectal cancer. Around 43% of patients showed elevated anti-MUC1 IgG levels following vaccination, and long-term memory was observed. T cell response and memory were also measured following a booster vaccine (107). Thus, this vaccine formulation could be useful for prophylactic vaccination in some patients with advanced adenomatous polyps (108).

A vaccine for patients with ductal carcinoma *in situ* (DCIS) has been developed to prevent progression. DCIS is often associated with HER-2/*neu* overexpression. Patients were given 4 doses of the Her-2/*neu*-pulsed dendritic cells. This vaccine resulted in lower HER-2/*neu* expression and T and B lymphocyte accumulation in the breast. Tumorlytic antibodies were observed. This vaccine lowered HER-2/*neu* expression in addition to decreasing residual DCIS following resection. These results suggest possible prophylactic value for this vaccine formulation (109).

### Virus-Like Particles

VLPs have been studied as a way to induce a combined humoral and cellular response. An mRNA-based VLP was developed to target prostate cancer, which has shown responsiveness to mRNA-based vaccines in previous studies (110, 111). Obtaining sufficient *in vivo* potency for nucleic acid vaccines has been difficult, as repeated use of viral vectors results in a dampened immune response (112). A recombinant bacteriophage MS2 mRNA-based VLP was developed using pESC yeast epitope tagging vectors, and PEG precipitation for synthesis to induce both a humoral and cellular response (12). VLP-vaccinated C57BL/6 mice exhibited elevated levels of IgG antibodies and increased antigen-specific cytotoxic T lymphocytes. Further investigation found that the initial Th2 response was converted to Th1 by target proteins. Mice were injected with TRAMP-C2 cells, a murine model of prostate cancer, and vaccinated mice were effectively protected from tumor development (12). This vaccine offers many advantages when compared to other nucleic acid vaccines, such as easy preparation using recombinant protein technology, and production of a strong humoral and cellular response.

### Carbohydrate-Based Vaccines

Carbohydrate-based vaccines have been engineered to induce a combined humoral and cellular response. A fluoro-substituted STn analogue was coupled with a nontoxic cross-reactive material of diphtheria toxin 107 (F-Stn-CRM197) for cancer prevention. When combined with Freund’s adjuvant, F-STn-CRM197 had significantly higher IFN-γ- and IL-4-releasing splenocytes compared to control. Vaccinated mice showed elevated levels of anti-STn IgG antibodies, which were further elevated with Freund’s adjuvant. The F-STn-CRM197 vaccine increased cellular and humoral immune responses when compared to a STn-CRM197 vaccine. This immune response resulted in increased cancer cell lysis. The data of Song et al. suggests the utility of this vaccine as a cancer prophylactic, building a basis for future carbohydrate-based cancer vaccine development (16).

### Autologous Tumor Cell Vaccines

Autologous tumor cell vaccines are derived from a patient’s own tumor, and although this personalized formulation has been exclusively therapeutic, it holds potential as building blocks for preventative vaccines (32). Agenus’ autologous tumor cell vaccine, AutoSynVax, has controlled tumor growth and produced lasting immune responses in recent pre-clinical and phase I clinical trials (34). While this vaccine is therapeutic, the development of autologous tumor cell vaccines to prevent recurrence is underway to prevent cancer recurrence in the setting of high-risk cancer patients. For instance, the use of autologous induced pluripotent stem cells (iPSCs) has been proposed for the development of an autologous tumor cell vaccine (33). Mice vaccinated with iPSCs combined with a CpG adjuvant were inoculated with B16F0 melanoma cells four weeks later. Vaccinated mice showed decreased tumor progression, and spleen analysis revealed increased tumor-specific effector and memory helper T cells. Additionally, there were more mature APCs found in the vaccinated group. While antibody analyses were not included in the study, increased IgG responses were measured in therapeutic experiments, indicating the possibility that this formulation could successfully induce both a humoral and cellular response. This study showed that prophylactic immunization with non-genetically engineered iPSC-based vaccines produce immune responses to melanoma. These vaccines have the potential for tumor immunity to a larger number of cancer types, which is supported by the large number of tumor antigens presented. Both humoral and cellular effects were observed. The use of autologous iPSCs was suggested as they may provide an accurate and personalized panel of a patient’s tumor immunogens (33).

### Allogenic Tumor Cell Vaccine

Allogenic tumor cell vaccines differ from autologous vaccines in that they are derived from another patient’s cells. Melacine, an allogenic tumor cell vaccine for treatment of melanoma, has undergone phase I, II, and III clinical trials, and survival benefits for patients were observed (35). The overall success of allogenic tumor cell vaccines has been limited to therapeutic immunotherapy (38). A preventative vaccine has been proposed using a vaccine derived from the fusion of allogenic DCs with tumor cells (36). DC-tumor fusion vaccines allow for the presentation of a broad spectrum of tumor-associated antigens (113). This vaccine was engineered using PEG-mediated fusion between DCs and inactive gastric cancer cells. The fused cell (FC) vaccine was combined with CTLs to prevent gastric cancer. Vaccinated mice showed slowed tumor growth compared to the control, with 9 of 10 remaining tumor-free and surviving for 90 days. The vaccine successfully induced cytotoxic T lymphocyte cloning through induction of antigenic determinants, resulting in anti-tumor effects. Furthermore, IL-7 and IL-15 levels increased following immunization, indicating immune memory formation.Elevated levels of IFN- γ and IL-10, which enhance B cell survival and antibody production, were observed. This study verified antigen-presenting and tumor-targeting effects from DC-based tumor vaccines and provides a template for future vaccine development (36).

Our group recently fabricated a preventative vaccine for triple-negative breast cancer (37). This vaccine was developed by sonicating 4T1 breast cancer cells and delivering the tumor nano-lysate (TNL) to BALB/c mice *via* tail vein injection 10 d before 4T1 tumor inoculation. Tumor growth and metastasis were significantly delayed, and survival was increased for mice in the vaccinated group compared to the unvaccinated group. While the TNL-vaccinated mice ultimately developed tumors, the success of this simple process motivates future studies to engineer similar vaccines to produce a preventative response (37).

### DNA Vaccines

The idea of DNA vaccines has received much attention over the past decade (114). A DNA vaccine to induce both cellular and humoral responses *in vivo* has been proposed to prevent HPV infection, specifically high risk HPV16 and 18, or HPV16-E7-expressing tumors (115). These viruses, encoding for oncoproteins E6 and E7, promote cervical cancer development (116). The proposed polynucleotide vaccine uses a designed DNA sequence coding for an E6/E7 fusion protein. Vaccinated mice had complete tumor prevention when injected with TC-1 cells, a tumor cell line derived from primary lung epithelial cells that are E6- and E7-expressing (117). Vaccination resulted in an E7-specific antibody response that lasted at least five months. E6- and E7-specific T cells could be identified after 5 months (115). Despite the many obstacles to successful DNA vaccines, this vaccine serves as evidence that the use of prophylactic cancer vaccines is possible and should be further studied (115).

## Conclusions

Preventative vaccines have helped to eradicate many diseases. Cancer remains one of the leading causes of death and healthcare burden in the United States. The development of prophylactic cancer vaccines has the potential to save lives and reduce healthcare costs by going beyond treating cancer to preventing it altogether. These vaccines are currently in a variety of development stages, from concept design and research, to implementation, and clinical practice.

Some of these vaccines produce humoral or cellular immune responses, with associated advantages and disadvantages. While, humoral vaccines allow for long-term immune protection and may be used to target secondary tumor antigens, B cell tolerance can limit their effectiveness. Cellular vaccines can have both preventative and therapeutic benefits, but T cell exhaustion is a common problem that needs to be addressed. Engineered vaccines that induce both humoral and cellular immune responses could represent an innovative solution to address these shortcomings. However, all cancer vaccines must consider age-related immune decline, a problem magnified by the elevated age distribution of cancer patients. Aging is associated with decreased B cell prevalence and potency, attenuating the effectiveness of humoral immune responses. Furthermore, multifactorial phenomenon, including changes to the thymus, cause a decrease in T cell reactivity, resulting in reduced cellular immunity. These challenges require new, innovative solutions. One possible solution is combining cancer vaccine administration with immune augmentation treatments. Vaccinated patients with high-risk of cancer development may be given continuing doses, with increasing frequency as they age.

Many strategies have been discussed in this review to prevent tumor development *via* cellular, humoral or a combined immune response. VLPs and carbohydrate-based vaccines have been designed to induce humoral responses or a combined humoral and cellular immune response. VLP vaccines are able to overcome B cell tolerance due to their multivalent display of self-antigens, but stability must be addressed. Carbohydrate-based vaccines, which have the advantage of targeting unique glycan markers, often show poor immunogenicity. Peptide vaccines and DNA vaccines are able to induce a cellular response or a combination of humoral and cellular. Peptide vaccines are usually engineered to have high stability against degradation *in vivo* and are easy to synthesize but suffer from inefficient immune responses. DNA vaccines are easy to produce and stable, but exhibit inadequate immunogenicity. mRNA vaccines have the potential for high potency but lack *in vivo* stability. Autologous and allogeneic tumor cell vaccines utilize both cellular and humoral immunity, but most current uses are therapeutic in nature. Maximizing the potential of the immune system may be necessary to successfully engineer preventative cancer vaccines, requiring the utilization of both humoral and cellular immunity. Further research into these strategies will lead to improved prophylactic cancer vaccines.

Despite the benefits of each type of vaccine, DNA and mRNA vaccines are garnering increased attention. With new technologies being developed, it seems that DNA and mRNA vaccines may offer the most promise for future research. DNA and mRNA vaccines may be developed to specifically target tumor antigens and promote specific immune responses against tumor onset. New technologies in LNPs and other nanomaterial carriers may help overcome stability problems associated with mRNA vaccines, increasing potency of the vaccine.

Currently, there are very few prophylactic cancer vaccines on the market. Gardasil and Cervarix prevent HPV, while Energix-B, Recombivax HB and Hiberix-B prevent HBV; these two viruses are commonly associated with cancer development (118). These vaccines have been highly successful, and the number of cervical cancer patients has decreased as vaccination has become more prevalent. Following these successful viral vaccines, there is great potential in preventing cancers caused by viruses, which account for 15% of all cancers. Development of vaccines to prevent the remaining 85% of cancer types is underway. However, significant obstacles remain in the development of vaccines for these cancers not caused by viruses. While vaccines against HPV and HBV stop cancer through viral protection, preventing cancers with no known viral etiology will be much more challenging. Researchers must identify viable targets, engineer successful delivery mechanisms, and find long-lasting immune effects. Many current technologies allow for preventative success in non-human tests, but one of the major problems will be finding solutions at clinically relevant doses. Further issues include prevention of immune response to self-antigens. This challenge may be overcome by identifying cancer-specific membrane expression or pre-malignant tumor properties to target. One possible way to overcome this issue is through targeting of neoantigens, a type of tumor-specific antigen, as they are recognized as non-self by the immune system (56). Another possible target includes tumor-associated antigens, although those are more difficult since they are also found in healthy cells. Finding possible targets to prevent tumor onset is critical for successful vaccine development for spontaneous cancers (**Table 2**).

**Table 2:** Summary of the most promising prophylactic cancer vaccine formulations and possible antigens and targets associated with each vaccine strategy.

Vaccine Strategy	Example Vaccines Developed	Antigens/Targets
Virus-like Particles	Cervarix and Gardasil – Commercially available vaccines for HPV (78) HER2-VLP Vaccine – Vaccine for HER2-Postive breast cancer (79) MS2 mRNA-based VLP – Vaccine for prostate cancer (12)	HER2 – protein (79)
Carbohydrate-based	4-KLH Vaccine – Vaccine for colorectal cancer targeting STn (84)	Sialyl-TN – oncofetal antigen (17)
Peptide	KRAS-targeting Peptide Vaccine – Vaccine for lung cancer (100) MUC1-poly-ICLC Vaccine – Vaccine for colorectral cancer (107)	KRAS – proto-oncogene (100)MUC1- glycoprotein and TAA (107)
DNA	RALA-pPSCA-loaded MNs – Vaccine for CRPC (26)HPV Polynucleotide Vaccine – Vaccine for HPV16 and HPV18 (115)	Ral-A – protein (26)E6/E7 – oncoprotein (116)
Tumor-derived Exosomes	RT-TEX Vaccine – Vaccine for mammary carcinoma (29)	Multiple targets (29)
mRNA	Nasal Encapsulated mRNA Vaccine – Vaccine encoding for tumor antigen (31) MS2 mRNA-based VLP – Vaccine for prostate cancer (12)	Multiple targets (12, 31)
Allogenic Tumor Cell	Tumor Nano-Lysate Vaccine – Vaccine for triple-negative breast cancer (37)	Multiple targets (37)

Despite these obstacles, current research points to vaccine strategies that could be viable for cancer prevention. Success in animal models offers a promising template for clinical development. Several strategies discussed in this review seem viable for future development; additional insights may come by engineering solutions that combine multiple approaches. However, the benefits of prophylactic vaccine development justify these difficulties. Prophylactic cancer vaccines could be administered to high-risk groups. For example, those with familial risk of triple-negative breast cancer would be ideal candidates for vaccination with a breast cancer vaccine. Patients with hereditary non-polyposis colorectal cancer (HNPCC), a genetic predisposition to colorectal cancer, would also make ideal candidates for vaccination. Successful development of prophylactic cancer vaccines will lead to new challenges: when to administer vaccines, ideal vaccine patients, and proper monitoring of vaccine success in patients. Eliciting strong and lasting immune response is critical for the success of prophylactic vaccine implementation. Furthermore, immune responses must be directed at targets unique to tumor cells during the early stages of carcinogenesis. Likely, vaccines with the most success will elicit both humoral and cellular responses as they work together to strengthen anti-tumor response upon tumor onset. This will include T cell memory, antibody generation, and possible responses by other immune cells, such as dendritic cells. Vaccines can be engineered to induce these responses. Successful immune induction will likely include engineered peptides or carbohydrates combined with stabilizing chemicals. Development and fabrication of both primary components and stabilizing chemicals, such as PEG, PADRE, or liposomal encapsulations, could lead to the prevention of spontaneous cancer formation.

With a foundation for preventative cancer vaccines established, and approved vaccines to prevent two cancer-associated viruses, there is hope that more types of cancer will be prevented by engineering vaccines to evoke a specific immune response. Targeting and promoting the adaptive immune system to respond to a preventative, anti-cancer vaccine will be crucial to the adoption of more successful, prophylactic cancer vaccines.

## Author Contributions

DC and JD wrote the article, and MK edited it. All authors contributed to the article and approved the submitted version.

## Conflict of Interest

The authors declare that the research was conducted in the absence of any commercial or financial relationships that could be construed as a potential conflict of interest.
